# Synchronous Primary Gallbladder Cancer and Ipsilateral Renal Cell Carcinoma: A Case Report

**DOI:** 10.7759/cureus.101579

**Published:** 2026-01-15

**Authors:** Salwan K Alobaidi

**Affiliations:** 1 Gastrointestinal Surgery, Paky Hospital, Erbil, IRQ

**Keywords:** cancer of gallbladder, difficult cholecystectomy, ipsilateral renal cancer, radical surgery, synchronous cancers

## Abstract

The synchronous occurrence of primary gallbladder carcinoma and ipsilateral renal cell carcinoma is exceptionally rare. Such presentations pose significant diagnostic challenges, particularly in differentiating synchronous primary tumors from metastatic disease. We report the case of a 57-year-old male patient who presented to the clinic in October 2025 with right upper quadrant abdominal pain and was found to have both gallbladder carcinoma and a right renal mass. Comprehensive imaging confirmed two distinct primary malignancies. The patient underwent laparoscopic radical cholecystectomy combined with radical right nephrectomy. A pathological analysis was performed, which showed that the process was gallbladder cancer and renal cell carcinoma. The patient is now under follow-up. This report highlights the importance of accurate diagnosis and multidisciplinary management of rare synchronous malignancies.

## Introduction

The co-occurrence of multiple primary malignant neoplasms in a single patient, particularly synchronous presentations, represents a rare yet increasingly recognized clinical phenomenon, with reported incidences ranging from 1% to 10% [1]. Synchronous tumors, by definition, are multiple independent malignancies detected concurrently or within a six-month interval of each other [1]. While the incidence of gallbladder carcinoma in conjunction with other primary malignancies varies, synchronous renal cell carcinoma remains an exceedingly rare finding, reported in approximately 0.10% of cases with a 2:1 male-to-female ratio [2]. This challenging scenario underscores the necessity for comprehensive diagnostic workups to identify potential secondary malignancies, as their presence significantly influences therapeutic strategies and prognostic outcomes [2]. Such cases, often termed multiple primary cancers, necessitate careful differentiation from metastatic disease, a distinction typically achieved through thorough histopathological analysis and advanced imaging techniques that confirm distinct origins and tumor types [3].

The current case report delineates the diagnostic and therapeutic complexities of multiple primary malignancies, often neglected in standard clinical evaluations. Its documentation enriches the scant body of literature on synchronous primary cancers, fostering heightened clinical awareness and diagnostic acuity in comparable scenarios. This report details a unique case of synchronously presenting gallbladder carcinoma and ipsilateral renal cell carcinoma, an association rarely documented in existing medical literature [1,4].

## Case presentation

A 57-year-old male smoker with a 30-pack-year history, hypertension, hyperlipidemia, and poorly controlled diabetes mellitus presented to the clinic in October 2025 with severe right upper quadrant abdominal pain of three days' duration, necessitating transfer to the emergency department. Three months prior, he had experienced mild right-sided abdominal pain that resolved spontaneously, whereas the current episode was severe and unrelenting. He denied fever, weight loss, urinary symptoms, family history of malignancy, or changes in appetite.

Physical examination revealed stable vital signs and tenderness in the right upper quadrant without rigidity. The patient had worked as a radiology technician for 20 years.

Laboratory findings showed mildly elevated liver enzymes, including aspartate aminotransferase (AST) 72 U/L, alanine aminotransferase (ALT) 71 U/L, alkaline phosphatase (ALP) 114 U/L, total bilirubin 1.7 mg/dL, and direct bilirubin 1.2 mg/dL. C-reactive protein (CRP) was 9 mg/L, glycated hemoglobin (HbA1c) was 11.8%, and the white blood cell count (WBC) was 7.7 × 10⁹/L; renal function was normal (urea 33 mg/dL, creatinine 0.87 mg/dL). The provisional diagnosis was acute cholecystitis. He was subsequently admitted for further diagnostic evaluation and management.

Abdominal ultrasonography revealed a distended gallbladder containing a vascularized intraluminal polypoid lesion, accompanied by sludge, small calculi, and pericholecystic edema. Additionally, a complex cystic-solid lesion suspicious for malignancy was identified in the upper pole of the right kidney.

**Figure 1:**
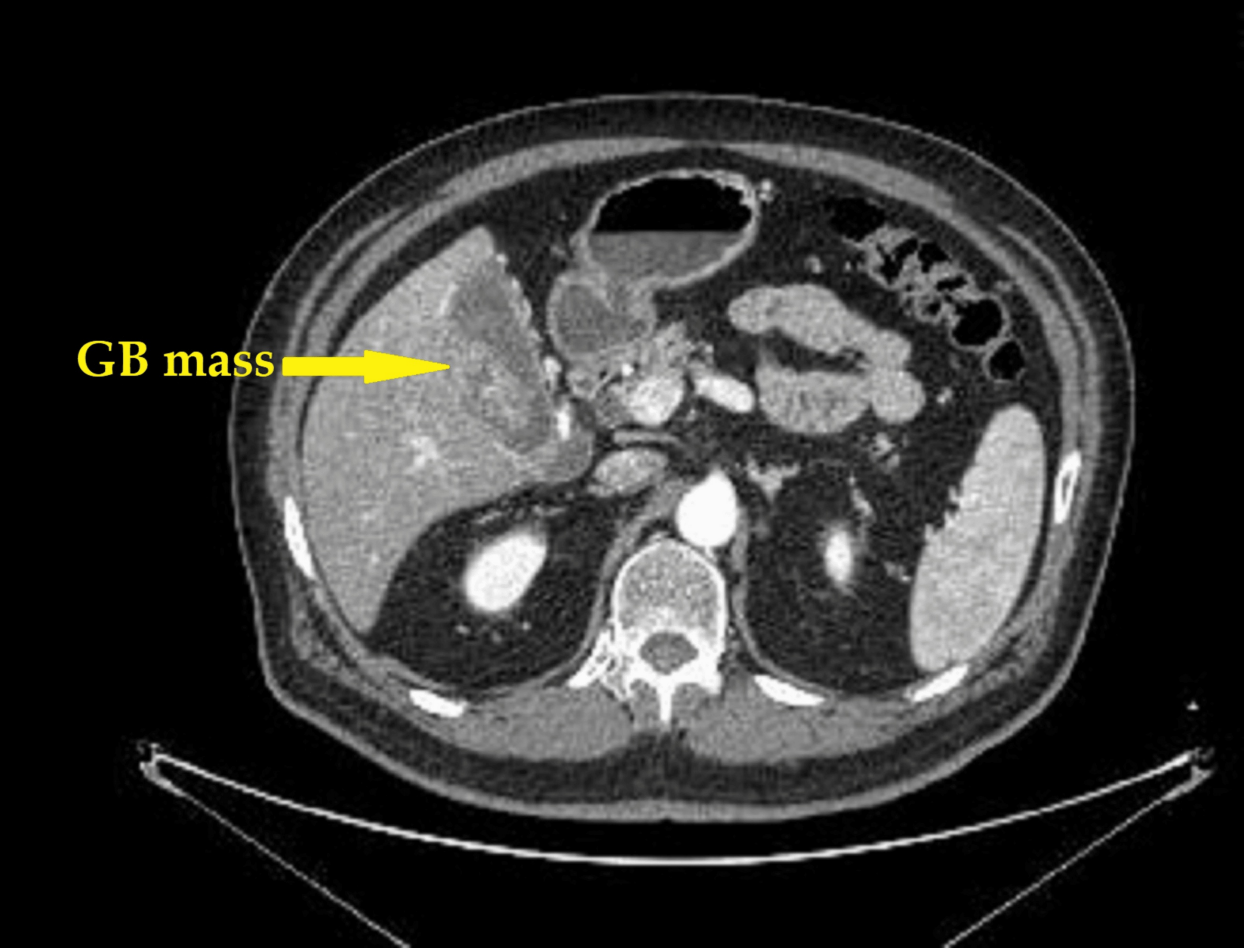
A CT scan showing a gallbladder mass (arrow) and an enlarged gallbladder (5*12.5 cm) in size showing multiple ill-defined, intensely enhancing, irregular masses; most likely gallbladder cancer.

**Figure 2:**
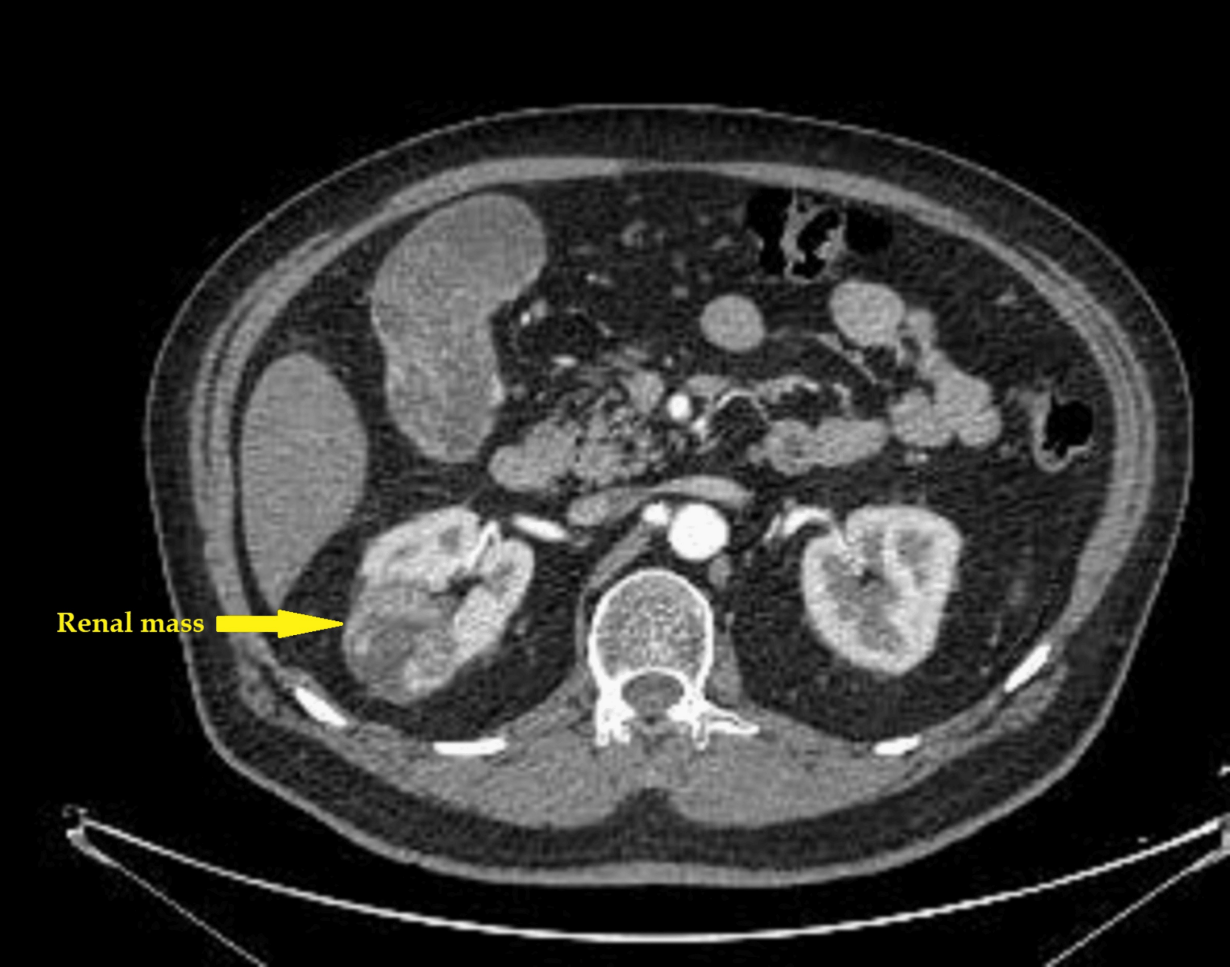
A CT scan showing a right renal upper pole mass (arrow), an inhomogeneous enhancing partially exophytic mass 43 mm in greatest dimension involving the posterior part of the upper pole. The nephrometry score was 8P, highly suggestive of renal-cell carcinoma.

Contrast-enhanced multidetector computed tomography [5] confirmed findings consistent with gallbladder carcinoma (Figure 1) and a right renal mass suggestive of renal cell carcinoma (Figure 2).

**Figure 3:**
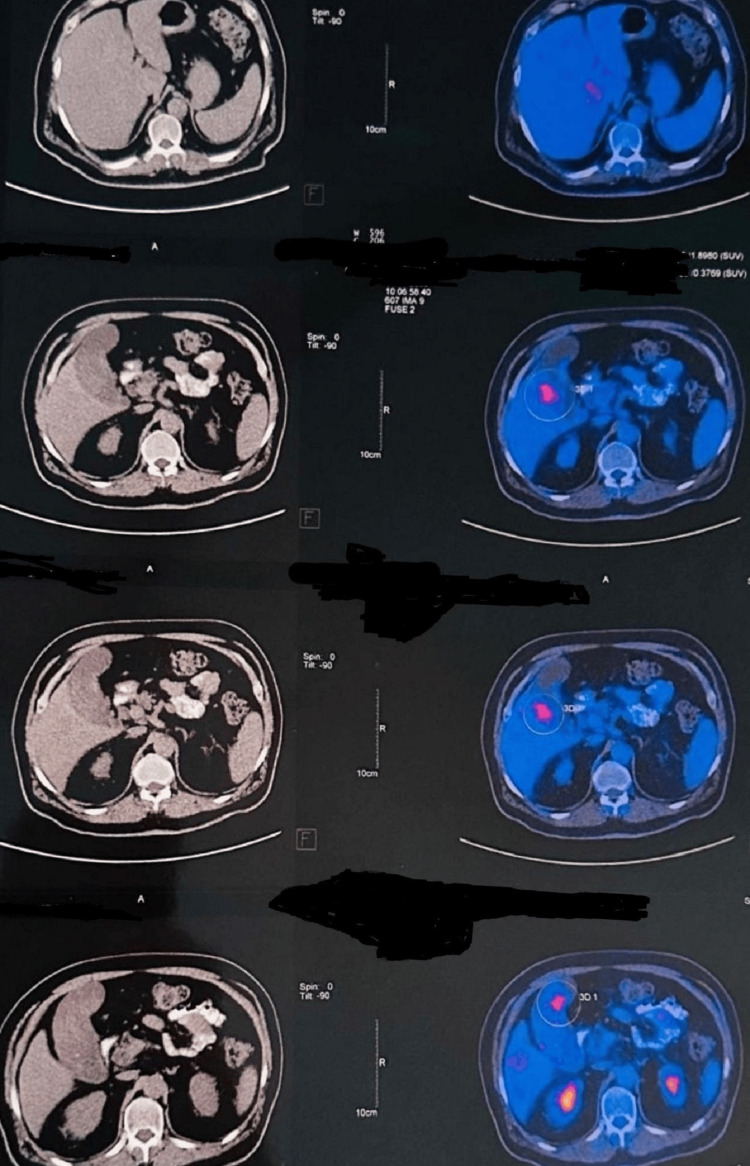
Positron emission tomography scan showing activity in gallbladder lesion (red spot inside the circle). A distended gallbladder with irregular wall thickening contains multiple small stones with at least three intramural tumor masses projecting into the lumen with no pericholecystic infiltration to adjacent liver.

**Figure 4:**
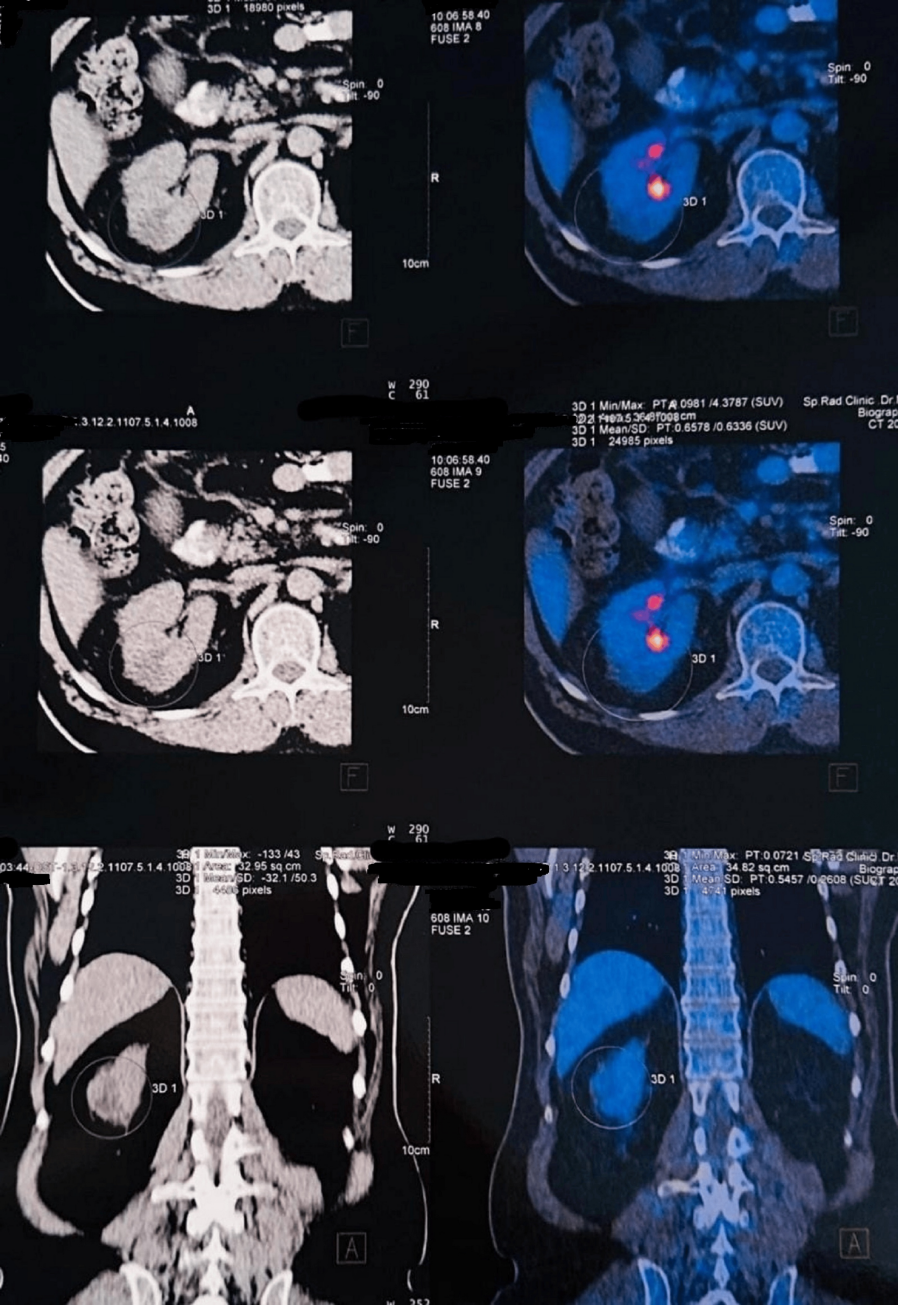
A PET scan of the right kidney showing cortical isodense, isometabolic (ISO) tracer uptake to normal renal cortex mildly bulging to the outside with low-density eccentric degeneration with an intact capsule and no evidence of locoregional lymphadenopathy (LAP).

Positron emission tomography demonstrated hypermetabolic activity limited to the gallbladder lesion and iso-tracer uptake in the renal mass, without evidence of distant metastasis (Figures 3, 4).

**Figure 5:**
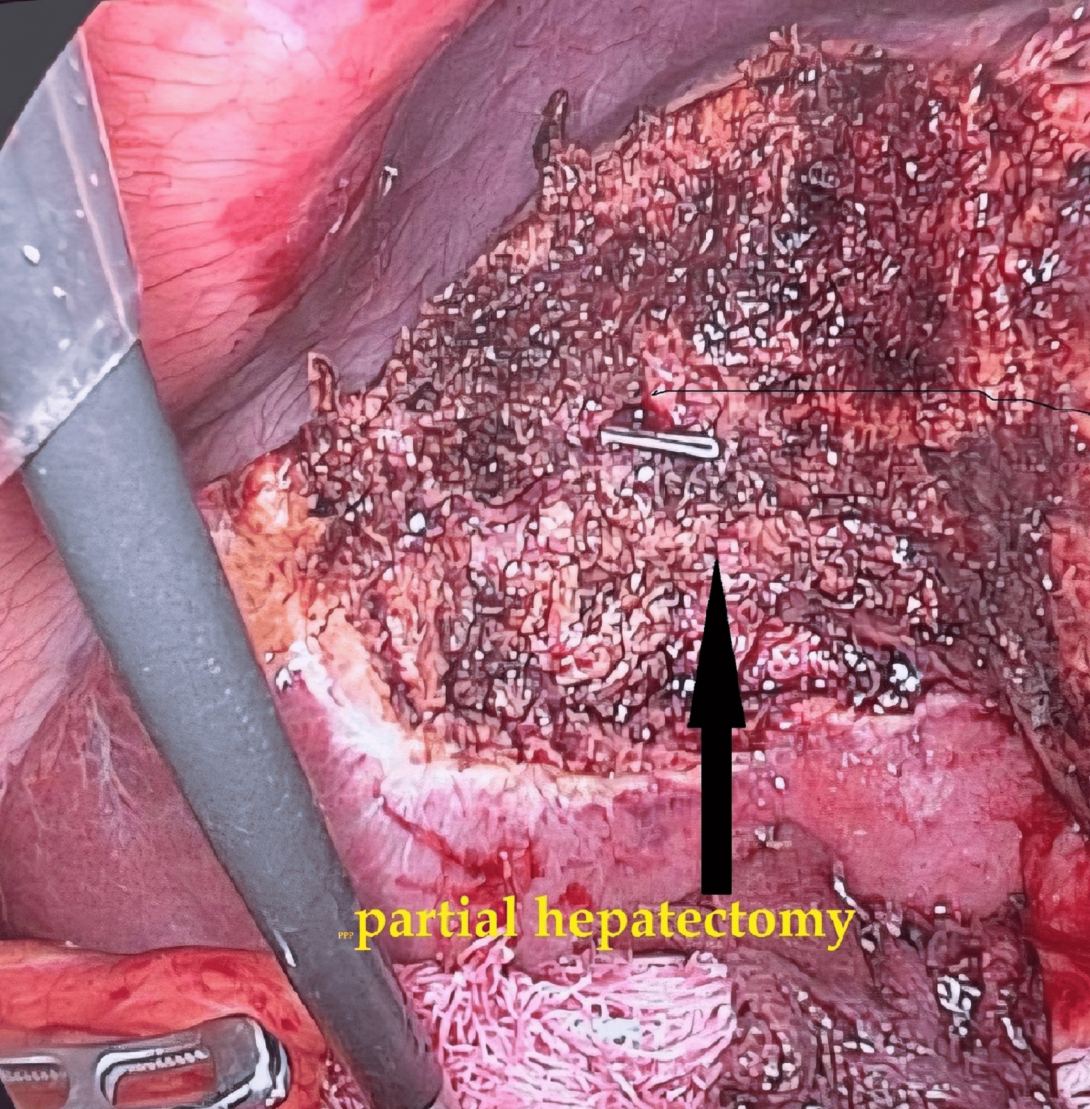
Intraoperative image of partial hepatectomy field (gallbladder removed and partial removal of liver tissue behind it).

**Figure 6:**
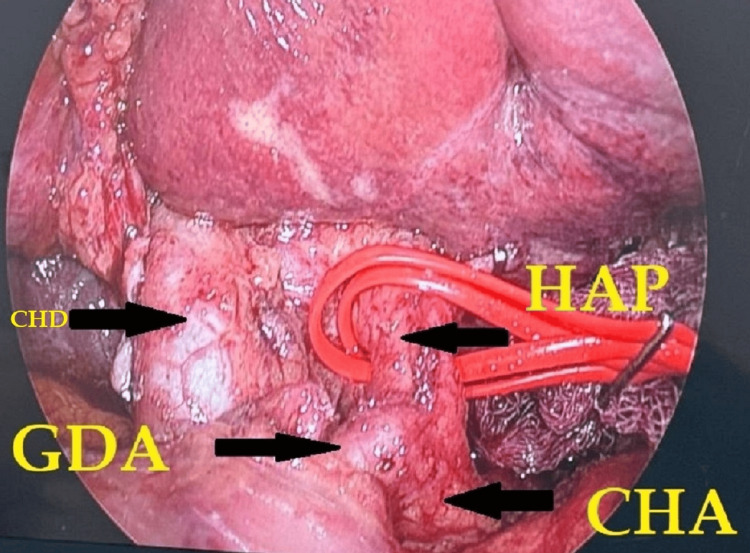
Laparoscopic dissection and porta hepatis lymphadenectomy HAP: hepatic artery proper; CHA: common hepatic artery; GDA: gastroduodenal artery; CBD: common bile duct

**Figure 7:**
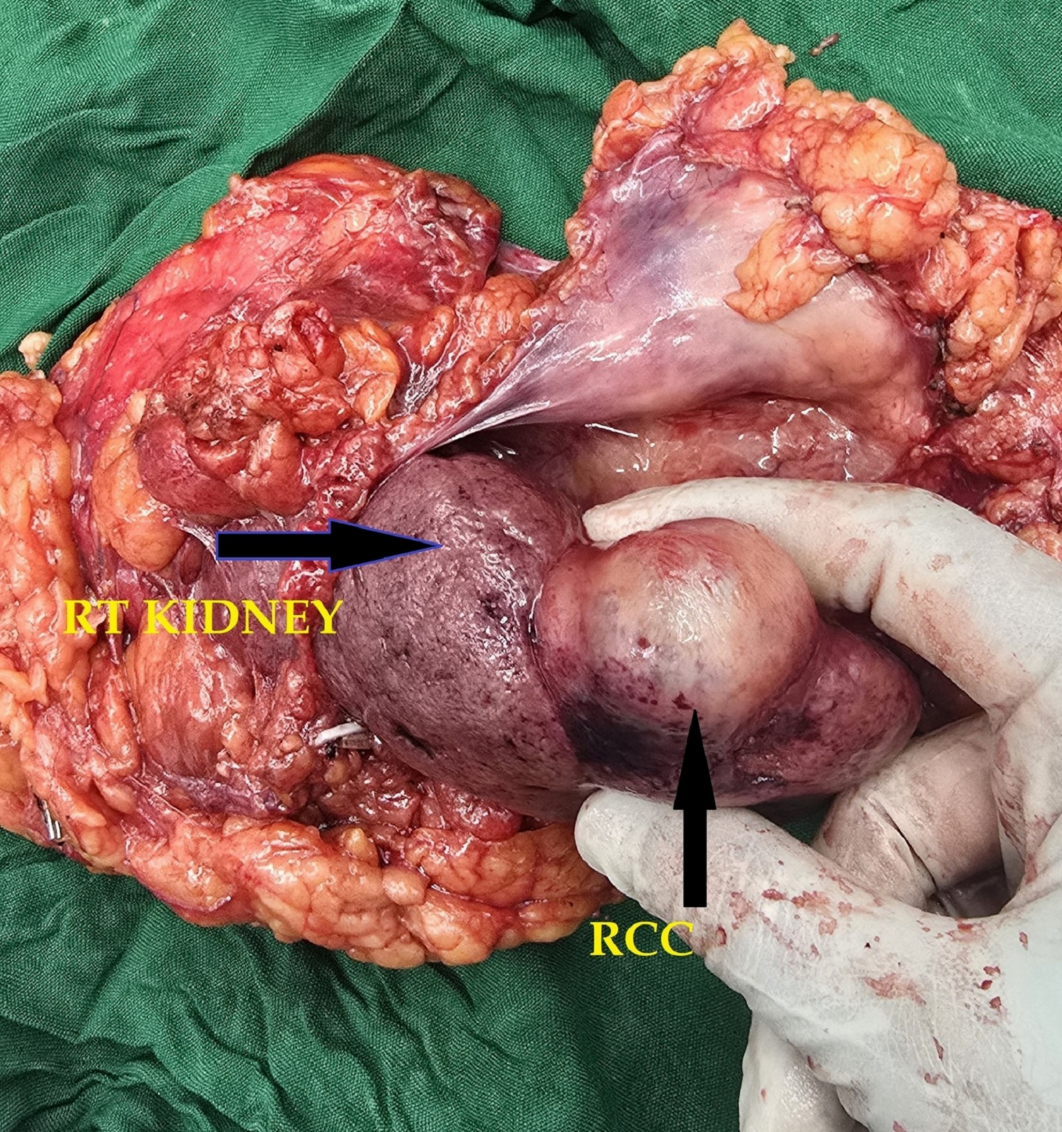
Surgical specimen showing right (RT) kidney and renal cell carcinoma (RCC).

**Figure 8:**
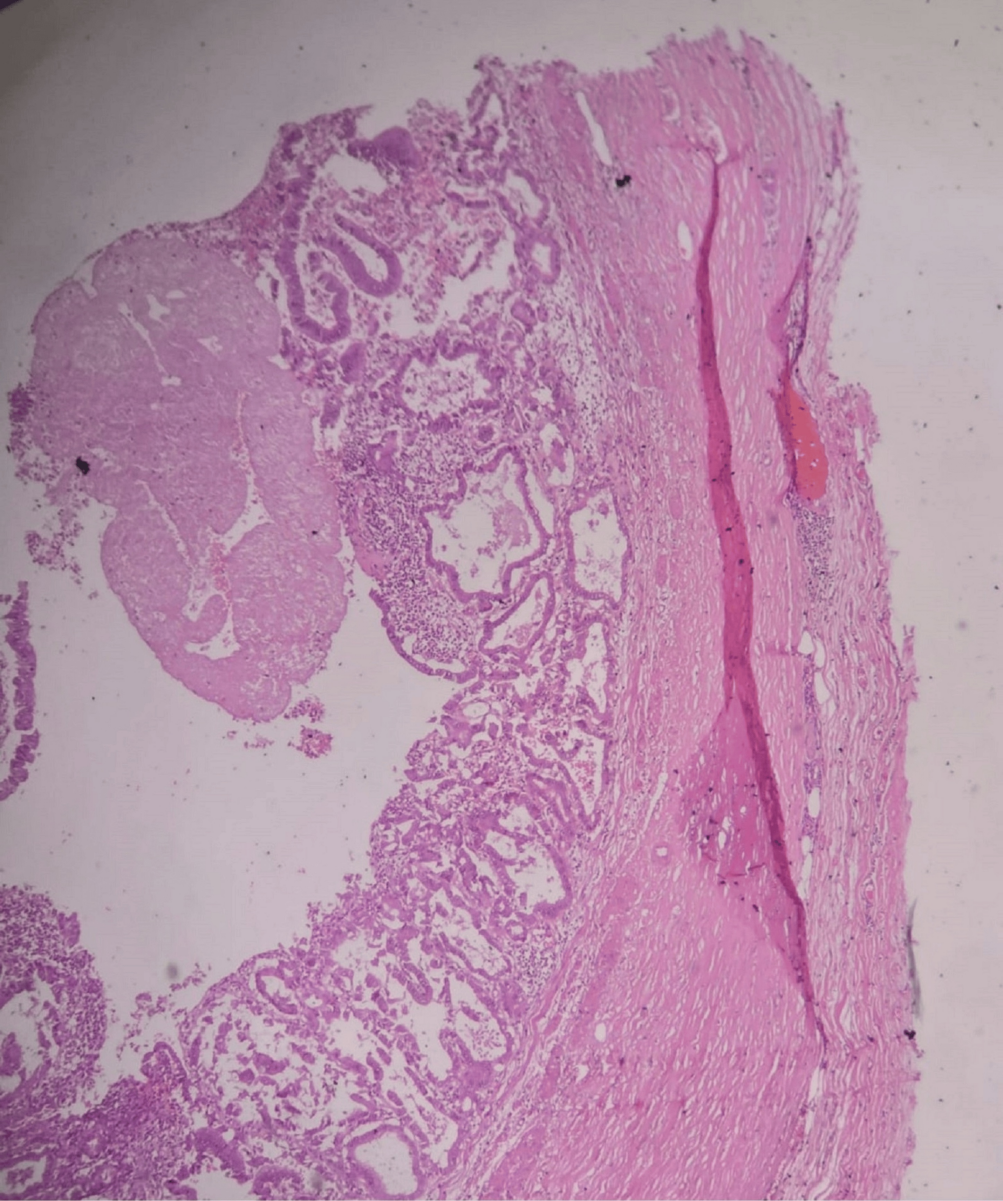
H&E stain showing the wall of gallbladder infiltrated by malignant glands (100X).

**Figure 9:**
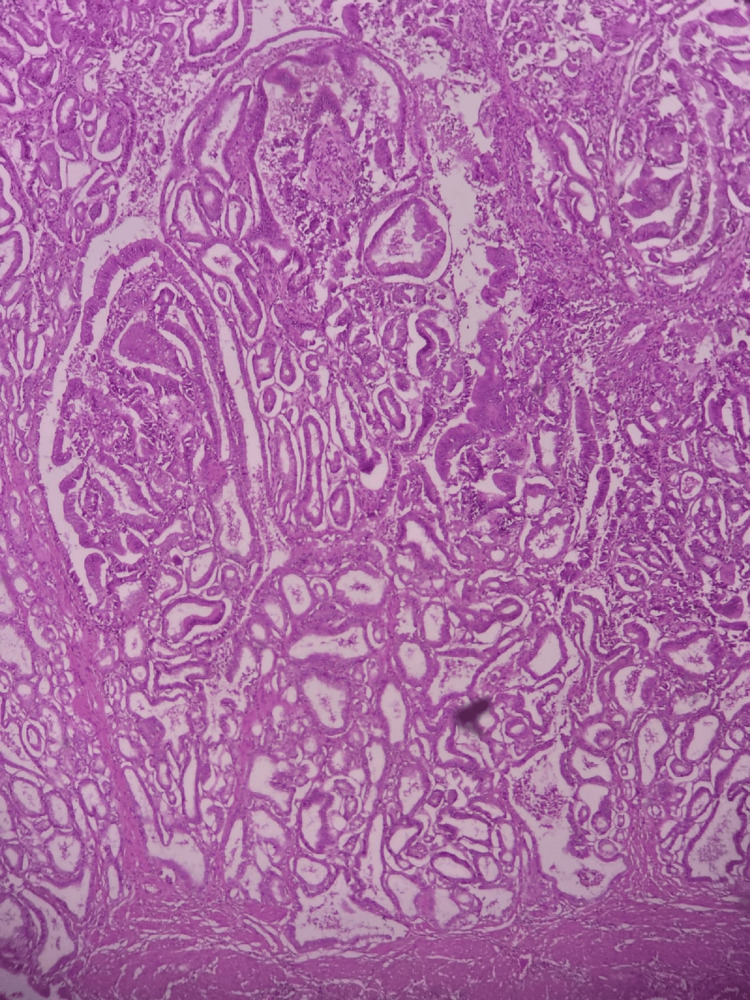
H&E stain showing complex glandular architecture protruding to the lumen and invading wall of gallbladder (100X).

**Figure 10:**
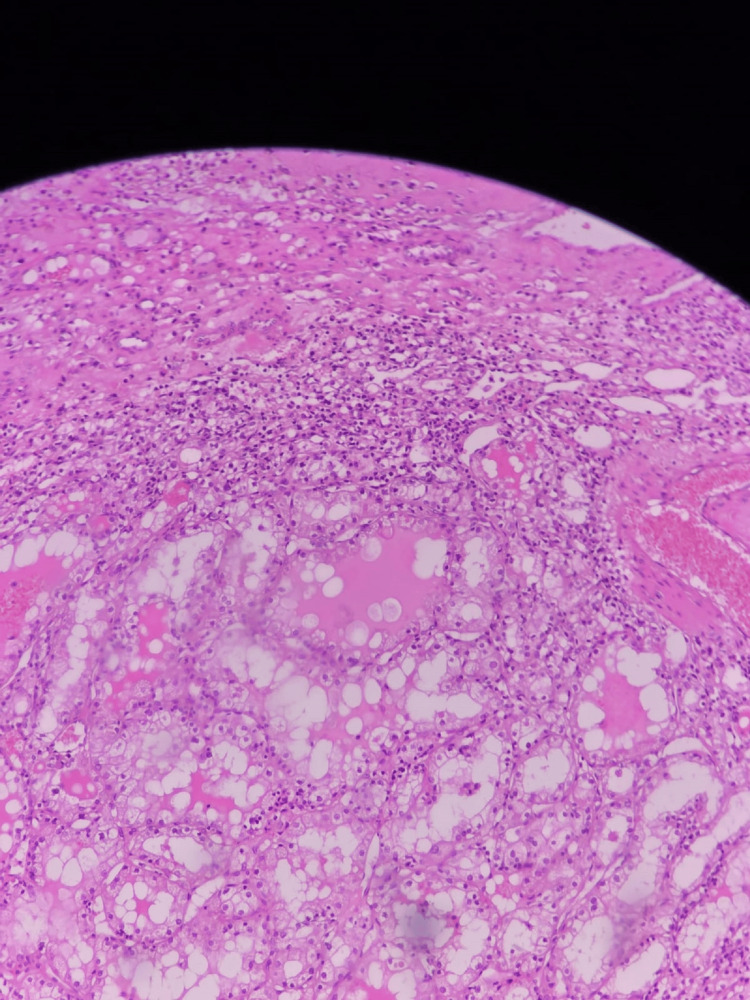
H&E stain showing renal cell carcinoma nests of cells with alveolar pattern and abundant clear cytoplasm (100X).

**Figure 11:**
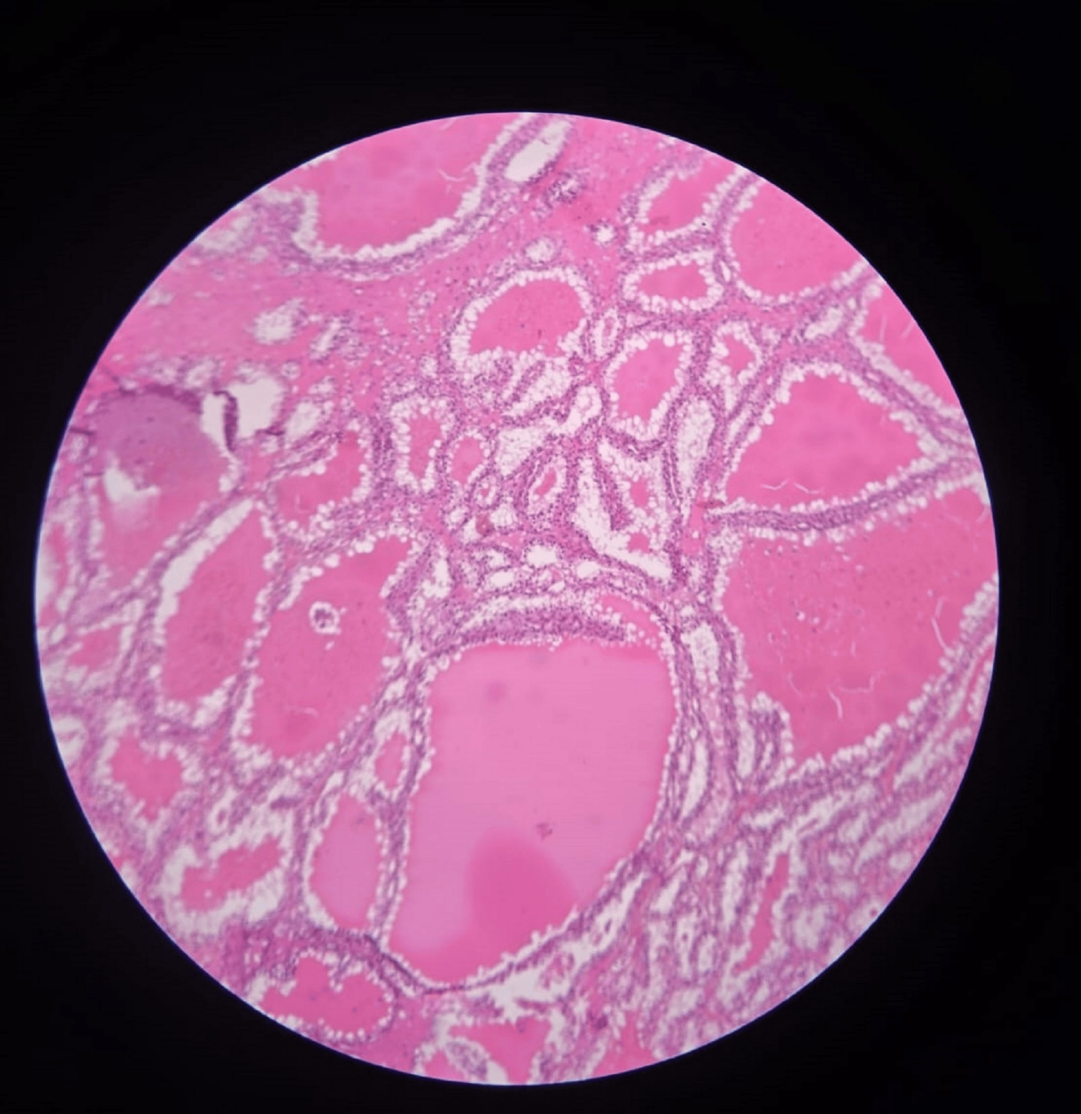
H&E stain showing renal cell carcinoma with tubular pattern of growth, nucleoli not seen in this power (100X).

Before surgery, the patient underwent multidisciplinary preoperative optimization involving endocrinology and cardiology consultations to improve glycemic control and cardiovascular readiness for the extensive procedure. After a urological assessment recommending radical right nephrectomy, the patient underwent a single-stage laparoscopic radical cholecystectomy incorporating partial hepatectomy (Figure 5) and porta hepatis lymphadenectomy (Figure 6), performed concurrently with radical right nephrectomy (Figure 7); the procedure lasted approximately eight hours. He was admitted postoperatively to the intensive care unit for close monitoring, remaining there for five days before transfer to the ward for three additional days, followed by discharge in stable condition. The postoperative course was uneventful, with ongoing follow-up. Histopathological examination confirmed primary invasive papillary adenocarcinoma (moderately differentiated, pathological stage T1aN0Mx) (Figures 8, 9) and clear cell renal cell carcinoma (pathological stage T1a) (Figures 10, 11). Following histopathological confirmation, an oncology consultation was obtained; the oncologist determined that adjuvant chemotherapy was not indicated.

## Discussion

This case highlights the diagnostic challenge of distinguishing between multiple primary malignancies and metastatic disease-a critical differentiation for guiding appropriate therapeutic interventions and predicting patient outcomes [1]. The rarity of such synchronous presentations underscores the necessity for clinicians to maintain a high index of suspicion, even in the absence of typical metastatic patterns, thereby ensuring thorough diagnostic workups that may unveil occult primary tumors [2].

The precise mechanisms driving synchronous development in anatomically distinct sites such as the gallbladder and kidney remain incompletely understood, though hypotheses often invoke genetic predispositions, shared environmental carcinogen exposures, or organ-specific risk factors. In this case, the patient's 20-year history as a radiology technician suggests potential chronic low-dose radiation exposure as a contributing factor for both malignancies. This contrasts with smoking and advanced age, which were highlighted as primary risks in the Martín-Pérez et al. series [2]; notable similarities include male predominance in renal cell carcinoma cases and the role of metabolic comorbidities, which may promote tumorigenesis via chronic inflammation or hormonal dysregulation, though these are not explicitly detailed in the reviewed literature. Moreover, the presentation of severe right upper quadrant pain due to gallbladder pathology, with incidental detection of an ipsilateral renal mass, parallels gastrointestinal symptoms and incidental findings in prior reports, underscoring how symptomatic primaries often unmask synchronous lesions.

This distinction is paramount for guiding therapeutic strategies, as the management of multiple primary malignancies often differs markedly from that of metastatic disease, which typically requires systemic rather than localized treatments [3]. The comprehensive multidisciplinary approach employed here highlights the critical importance of team-based care in managing complex synchronous primary malignancies, particularly given the distinct biological behaviors and treatment paradigms of gallbladder carcinoma and renal cell carcinoma [5-7]. Such collaboration is pivotal for optimizing patient care pathways, especially when surgical interventions involve extensive resections that demand meticulous preoperative planning and postoperative management.

Furthermore, the absence of adjuvant chemotherapy in this case-despite two distinct primary malignancies-emphasizes the individualized nature of oncological treatment strategies, which are often dictated by specific pathological findings and patient-specific factors rather than universal protocols [8]. This decision aligns with current guidelines prioritizing personalized medicine, particularly for synchronous rare cancers where the efficacy of systemic adjuvant therapy remains largely undefined [9]. Limited data on follow-up and treatment outcomes for such malignancies further complicates standardized adjuvant protocols, necessitating a cautious, evidence-based approach tailored to each patient's unique clinical profile [10].

The prevalence of multiple primary malignancies, ranging from 0.73% to 11.7%, necessitates meticulous diagnostic approaches to differentiate synchronous tumors (diagnosed within a six-month interval) from metachronous occurrences or metastatic disease [11, 12].

## Conclusions

In conclusion, the simultaneous presentation of primary gallbladder adenocarcinoma and ipsilateral clear cell renal cell carcinoma exemplifies a rare and diagnostically challenging clinical scenario that necessitates a comprehensive multidisciplinary approach to optimize patient management and treatment outcomes. The successful single-stage surgical resection and uneventful postoperative recovery detailed in this report underscore the feasibility of radical procedures for synchronous primary malignancies, predicated on meticulous preoperative optimization and interdisciplinary collaboration.

## References

[1] Yavuz Onur Danacıoğlu, Ferhat Keser, Pınar Engin Zer (2019). Synchronous tumors: renal cell carcinoma with adenocarcinoma of the ampulla of Vater; case report.. Medeniyet Med J.

[2] Martín-Pérez JA, Torres-Silva C, Tenorio-Arguelles R (2020). Gastric carcinoma and renal cell carcinoma as an atypical presentation of multiple primary malignancies: a case report and review of the literature. J Med Case Rep.

[3] Wu ZJ, Wang B, Zhao SC, Pan ZT (2025). Synchronous cholangiocarcinoma and cervical squamous cell carcinoma managed via a multidisciplinary approach: a case report. World J Clin Oncol.

[4] Ozkanli S, Turfanda EA, Zenginkinet T, Aydin A (2014). Cooccurrence of multilocular cystic renal cell carcinoma and high-grade urothelial carcinoma in bladder. J Med Cases.

[5] Umemura A, Nitta H, Takahara T (2020). Identifying cystic vein perfusion area employing indocyanine green fluorescence imaging during laparoscopic extended cholecystectomy for clinical T2 gallbladder cancer. Case Rep Gastroenterol.

[6] Kinoshita O, Dohi M, Horii Y, Ikai A, Kitamori T, Yamashita T (2019). Simultaneous resection of gastric and gallbladder metastasis from renal cell carcinoma treated by laparoscopic and endoscopic cooperative surgery: a case report. Surg Case Rep.

[7] Fraga J, Caetano Oliveira R, Alexandrino H, Cipriano MA (2019). Neuroendocrine carcinoma and intracystic papillary neoplasm: a rare association in the gallbladder. GE Port J Gastroenterol.

[8] Babarasul MH, Bapir R, Rahman DH (2023). Synchronous ipsilateral papillary renal cell carcinoma and urothelial carcinoma: a case report. Oncol Lett.

[9] Sakr M, Badran M, Hassan SA, Elsaqa M, Elwany MA, Deeb NM, Sharafeldeen M (2024). Detection of two synchronous histologically different renal cell carcinoma subtypes in the same kidney: a case report and review of the literature. J Med Case Rep.

[10] Ünver M, Öztürk Ş, Bozbıyık O (2014). Incidental early gallbladder carcinoma with hepatocellular carcinoma: a rare synchronous double primary malignant tumor. J Med Cases.

[11] Zhang ZG, Chen Y, Ji R (2018). Synchronous cancers of gallbladder carcinoma and combined hepatocellular cholangiocarcinoma: an unusual case and literature review. BMC Cancer.

[12] Maria Leitão, Tiago Alpoim, Manuela Machado (2019). Multiple synchronous tumours: a peculiar clinical case. J Cancer Therapy.

